# Thyroid Hormone in the Clinic and Breast Cancer

**DOI:** 10.1007/s12672-018-0326-9

**Published:** 2018-02-13

**Authors:** Aleck Hercbergs, Shaker A. Mousa, Matthew Leinung, Hung-Yun Lin, Paul J. Davis

**Affiliations:** 1 Department of Radiation Oncology, Cleveland Clinic, Cleveland, OH USA; 2 Pharmaceutical Research Institute, Albany College of Pharmacy and Health Sciences, Albany, NY USA; 3 Department of Medicine, Albany Medical College, Albany, NY USA; 4 Program for Cancer Molecular Biology and Drug Discovery, College of Medical Science and Technology, Taipei Medical University, Taipei, Taiwan; 5 Taipei Cancer Center, Taipei Medical University, Taipei, Taiwan

## Abstract

There is preclinical and recent epidemiological evidence that thyroid hormone supports breast cancer. These observations raise the issue of whether management of breast cancer in certain settings should include consideration of reducing the possible contribution of thyroid hormone to the advancement of the disease. In a preliminary experience, elimination of the clinical action of endogenous L-thyroxine (T_4_) in patients with advanced solid tumors, including breast cancer, has favorably affected the course of the cancer, particularly when coupled with administration of exogenous 3,5,3′-triiodo-L-thyronine (T_3_) (euthyroid hypothyroxinemia). We discuss in the current brief review the possible clinical settings in which to consider whether endogenous thyroid hormone—or exogenous thyroid hormone in the patient with hypothyroidism and coincident breast cancer—is significantly contributing to breast cancer outcome.

## Introduction

Recent epidemiologic studies have described associations between thyroid function and breast cancer [1–3] that indicate the hormone can support this form of cancer. Clinical evidence is also available that hypothyroidism beneficially affects the course of breast cancer [4, 5]. We have shown that the hormone, specifically L-thyroxine, T_4_, is a proliferative factor in vitro for breast cancer cells [6] and that, in the absence of estrogen, thyroid hormone can promote nuclear estrogen receptor-α (ERα)-dependent proliferation of breast cancer cells bearing this receptor [6]. This effect involves mitogen-activated protein kinase (MAPK)-dependent protein kinase and activation of ERα by specific serine phosphorylation. The action of thyroid hormone in this setting we now know is mediated by a thyroid hormone receptor on the extracellular domain of integrin αvβ3 expressed by tumor cells [7, 8]. Thus, an action of thyroid hormone initiated nongenomically culminates in activity in the genomic pathway of ER action, an endocrine overlap that we have reviewed elsewhere [9]. The ER degrading agent, fulvestrant (ICI 182,780), blocks the proliferative action of thyroid hormone on ERα-positive human breast cancer cells [6]. It should be noted that L-thyroxine (T_4_) at physiological free hormone concentrations is maximally active at this receptor site on αvβ3 [10, 11], whereas substantially higher than physiological levels of free T_3_ are required to achieve substantive proliferative activity via this receptor.

We have also shown that the thyroid hormone receptor on αvβ3 enables triple-negative breast cancer cells to respond differentially to T_4_ in physiological concentrations [12] and to a T_4_ derivative and antagonist, tetraiodothyroacetic acid (tetrac), with changes in transcription of a panel of cell survival genes, including those for anti-apoptosis functions, for support of angiogenesis, and for chemoresistance [8, 12–14]. Breast cancer cells lacking the estrogen receptor proliferate in response to thyroid hormone via αvβ3 [13]. Thus, thyroid hormone is capable of supporting breast cancer cells by two mechanisms, depending upon the absence or presence of ER. We have reported clinically that a variety of far-advanced solid tumors, including breast cancer, stabilize or are reduced in size when patient T_4_ is eliminated pharmacologically and replaced with 3,5,3′-triiodo-L-thyronine (T_3_) (euthyroid hypothyroxinemia) [15].

In the current brief review, we discuss the possibility that endogenous physiologic range T_4_ in breast cancer patients or replacement T_4_ in hypothyroid patients who have coincident breast cancer, may increase the risk of continuing/recurrent tumor. As noted above, there are ER-dependent and ER-independent mechanisms that are relevant to both premenopausal and menopausal women with breast carcinoma. Acting via the αvβ3 integrin and independently of ERα, T_4_ serves to support tumor cell proliferation and the cell survival functions listed above [8, 12–14]. Then, regardless of the menopausal status of the ER-positive patient, thyroid hormone may stimulate ER-dependent breast cancer cell proliferation. Induction of euthyroid hypothyroxinemia has conceptual attractiveness in breast cancer, regardless of ER status of the tumor.

## Possible Actions of Circulating Endogenous T_4_ Within the Physiologic Range on Recurrent ERα-Positive Breast Cancer in the Pre- and Postmenopausal Patient

The therapy of recurrent ERα-positive breast cancer in premenopausal and postmenopausal women may include surgery of the primary or metastatic lesions, radiation, chemotherapy, and ER-targeted therapy, despite the minimized production of estrogen in the postmenopausal patient. Fulvestrant and tamoxifen are ER-targeted therapeutic agents in the setting of breast cancer. Direct comparisons of the effectiveness of these agents in breast cancer management are few in number and do not reveal clear-cut therapeutic advantages to monotherapy with either agent [16, 17]. Fulvestrant leads to degradation of ER, whereas tamoxifen stabilizes the receptor [18] and we propose that this distinction has implications for the action of T_4_ on ER-positive tumors. Eliminating ER with fulvestrant in ERα-positive tumors means that endogenous thyroid hormone cannot substitute for estradiol as a tumor-support agent. In contrast, T_4_ does not compete with steroids for the binding site on ER, but instead T_4_ activates MAPK to phosphorylate ER and thus we anticipated that tamoxifen would not oppose the proliferative action of T_4_ in ER-positive breast cancer. Preliminary studies, however, revealed that tamoxifen is capable of inhibiting the phosphorylation/activation of MAPK by T_4_ in breast cancer cells [19]. This novel action of tamoxifen on ER activation by T_4_ means that tamoxifen and fulvestrant are both capable of opposing the support of T_4_ for ER-positive cancer cells.

Against this background, we can ask whether tamoxifen in the pre- and postmenopausal euthyroid patient with ER-positive breast cancer has satisfactorily *eliminated* the possible contribution of thyroid hormone to tumor support. This issue has not been explored clinically or in animal models and does, in our opinion, require initial examination in the nude mouse bearing ER-positive human breast cancer xenografts that are exposed to several concentrations of tamoxifen. As noted above, we have shown fulvestrant to be effective anti-thyroid hormone therapy in MCF-7 breast cancer cells [6], but this requires confirmation in other ER-positive cell lines and primary cultures of breast cancer cells. Until more information on this issue is available, we propose in the patient whose ER-positive cancer remains active on regimens that include anti-ER treatment, that elimination of host T_4_ (euthyroid hypothyroxinemia) be considered as an option [15].

Long-term tamoxifen therapy (6 months) may alter circulating levels of thyroid hormone and TSH [20], increasing TSH and total T_4_ and T_3_, but not free hormone levels. In the absence of changes in free T_4_, it is unlikely that this tamoxifen effect is relevant to thyroid hormone actions on breast cancer.

## Possible Actions of Endogenous T_4_ Within the Physiologic Range on ERα-Negative Breast Cancer

We have shown that proliferation of ERα-*negative* human breast cancer cells may be induced by T_4_ via an integrin αvβ3-dependent mechanism [8, 12]. Tetrac, a specific pharmacologic inhibitor of T_4_ at integrin αvβ3, has expanded our understanding of the function of this receptor in cancer cells, notably, ER-negative breast cancer cells [13].

Are the distinctive actions of T_4_ on support of ER-positive and ER-negative breast cancer mutually exclusive? That is, if the pro-tumor contribution of T_4_ can be satisfactorily eliminated by fulvestrant in the ER α-positive breast cancer patient, will the proliferative effect of T_4_ become apparent via the alternative αvβ3 pathway? In acute in vitro studies of fulvestrant, no such activity was apparent [19], but longer term studies are required.

## Consideration of Induction of Euthyroid Hypothyroxinemia in the Setting of Breast Cancer

As noted above, there is clinical evidence that hypothyroidism may beneficially influence the course of breast cancer [1, 4, 5]. Thus, in the setting of recurrent breast cancer post-standard therapeutic interventions, we suggest that induction of euthyroid hypothyroxinemia is an alternative.

However, there is no systematic prospective study available to support this intervention, nor is there clinical information to support elimination of host T_4_ at the point of initial tumor diagnosis and in conjunction with standard initial therapy. In the setting of induced euthyroid hypothyroxinemia in cancer patients, there is no clinical information available about the pharmacokinetics and pharmacodynamics of standard chemotherapeutic treatments of breast cancer that might be altered with the administration of replacement T_3_ two or three times daily.

There is another possible advantage of euthyroid hypothyroxinemia, namely, that elimination of the action of T_4_ at integrin αvβ3 may serve to radiosensitize cancer cells [21, 22]. We have shown that the T_4_ analogue and antagonist, tetrac, impairs tumor cell repair of radiation-induced double-stranded DNA breaks [22] in glioblastoma and prostate cancer cell lines, but this property has not yet been studied preclinically in breast cancer cells.

## Effect of Endogenous or Replacement T_4_ Within the Normal Range on Risk of Development of Breast Cancer; T_3_ and Breast Cancer

Certain epidemiologic studies have documented that circulating levels of T_4_ in the upper quartiles of the normal range are a risk factor for development of certain cancers, including breast [1, 2]. If this is indeed the case, then as an outgrowth of the discussions above, should primary care physicians monitor serum thyroid hormone levels in all women patients as a breast cancer risk factor and concentrate breast cancer screening efforts in patients with thyroid hormone levels in the upper one-half of the normal range? Such a risk analysis has not been conducted.

This possible risk issue applies additionally to patients with primary hypothyroidism who are on T_4_ as thyroid hormone replacement. The 2014 guidelines of the American Thyroid Associations for the treatment of hypothyroidism do not consider management of patients with hypothyroidism and nonthyroidal cancer [23]. Based on epidemiologic data currently available and cited above, thyroid hormone replacement treatment goals might include circulating thyroid hormone levels that are in the lower quartiles of the normal range. This T_4_-based treatment strategy may not be compatible with relief of symptoms of hypothyroidism in certain patients, and the strategy has not been tested in the hypothyroid-breast cancer patient population.

An alternative approach to be tested is T_3_ replacement. As noted above, the thyroid hormone receptor on integrin αvβ3 that is generously expressed by cancer cells and not by infrequently dividing nonmalignant cells is primarily a binding site for T_4_ [7, 8]. At this site, T_4_ supports breast cancer [6, 24]. Limited clinical evidence indicates that replacing systemic T_4_ with T_3_ retards breast cancer growth and growth of other solid tumors [15]. A number of preclinical studies also suggest that T_3_ retards breast cancer cell proliferation [25–27], but supraphysiologic concentrations of T_3_ were often involved in such studies. Additional clinical studies are needed of the possible therapeutic contribution of T_3_ to management of breast cancer and other solid tumors.

## Is the Level of Circulating Endogenous T_4_ a Risk Factor for Development of Breast Cancer in Euthyroid Patients with Positive Family Histories of This Form of Cancer?

This possibility has not been examined epidemiologically. The epidemiologic data cited above that attribute cancer risk to upper-quartile-of-the-normal-range thyroid hormone levels were not designed to examine familial disease.

## In Patients with Breast Cancer and Nonthyroidal Illness Syndrome, Is Elevated Circulating Free T_4_ and/or Total T_4_ Maintained Within the Normal Range a Factor Contributing to Breast Cancer Outcome?

Elevated free T_4_ levels in nonthyroidal illness syndrome (NTIS) are usually transient and related to acute or subacute inflammatory illness, rather than to chronic illness and cancer [28]. Thus, encounters with elevated free T_4_ levels in patients with cancer of any type are infrequent. However, endogenous total T_4_ levels may be maintained within the normal range by cancer patients who express NTIS via the hallmark of decreased circulating T_3_ concentration. In this setting, we raise for discussion the possibility that the persistence of aggressive cancer behavior in NTIS patients may be in part attributable to host T_4_ levels [19].

## Summary

T_4_ functions as a prohormone for T_3_ when genomic actions of thyroid hormone that depend upon the thyroid hormone nuclear receptor are under consideration. By discrete molecular mechanisms that originate at the thyroid hormone receptor on integrin αvβ3 and are ERα-dependent or independent of ER, thyroid hormone as T_4_ can support breast cancer. In this setting, T_4_ is a hormone, not a prohormone. The affinity of the hormone receptor on the integrin is higher for T_4_ than for T_3_, and it is physiological concentrations of free T_4_ that we have shown to stimulate human breast cancer cell line proliferation. As noted in this review, limited clinical and preclinical evidence indicates that T_3_ may arrest breast cancer growth. Preclinical data may reflect supraphysiological levels of T_3_, however, and such levels in certain in vitro systems have been reported to stimulate breast cancer cell proliferation [25].

The current review emphasizes that we do not yet know enough about the significance of contributions of T_4_ to breast cancer behavior. We do need clinical studies to assess prospectively the possible contribution of endogenous T_4_ or replacement T_4_ to aggressive behavior of ERα-positive and ERα-negative breast cancer in populations both of pre- and postmenopausal patients. What is to be sought are direct correlations between levels of the hormone within the normal range and cancer behavior and, where relevant, in the absence and presence of anti-ER therapy with fulvestrant or tamoxifen. If such documentation is obtained, then the utility of pharmacological manipulation of physiologic range T_4_ in clinically active breast cancer becomes apparent. We do not know enough about the actions of T_3_ on breast cancer behavior, although euthyroid hypothyroxinemia—a therapeutic setting in which T_3_ replaces host circulating T_4_—has been shown in limited clinical examination [14] to achieve arrest of growth in the case of breast cancer and other solid tumors.

The evidence we have reviewed here does not suggest that thyroid hormone causes breast cancer.
